# Antibacterial Activity and Toxicity of Zinc Oxide Nanoparticles Combined with Supernatants of *Lactobacillus* spp. Against ESKAPE Bacteria: A Novel Mixture

**DOI:** 10.5812/ijpr-139222

**Published:** 2023-12-02

**Authors:** Somayeh Soleymanzadeh Moghadam, Reza Hosseini Doust, Ali Majidpour, Mahdi Adabi, Sara Minaeian

**Affiliations:** 1 Department of Microbiology, Faculty of Advanced Science and Technology, Tehran Medical Sciences, Islamic Azad University, Tehran, Iran; 2 Antimicrobial Resistance Research Center, Institute of Immunology and Infectious Diseases, Iran University of Medical Sciences, Tehran, Iran; 3 Department of Medical Nanotechnology, School of Advanced Technologies in Medicine, Tehran University of Medical Sciences, Tehran, Iran

**Keywords:** *Lactobacillus acidophilus*, ESKAPE Pathogens, *Lactobacillus plantarum*, Zinc Oxide Nanoparticles

## Abstract

**Background:**

The emergence of multidrug resistance among nosocomial pathogens has prompted researchers to look for new antibacterial sources. Metal nanoparticles and probiotic products have attracted the attention of researchers. However, combination therapy is an attractive alternative in this field.

**Objectives:**

This study evaluated the antibacterial activity and toxicity of Zinc Oxide nanoparticles (ZnO-NPs) combined with cell-free supernatant (CFS) of *Lactobacillus plantarum* and *Lactobacillus acidophilus* alone and in a novel mixture.

**Methods:**

Antibacterial effects and cytotoxic properties of ZnO-NPs, CFS of *L. plantarum* (SLP), and CFS of *L. acidophilus *(SLA) were determined alone and in a mixture against ESKAPE strains. In addition, the viability percentage of the cells was evaluated after exposure to these agents.

**Results:**

Antibacterial mixtures (ZnO-NPs with SLP or ZnO-NPs with SLA) demonstrated synergistic and additive effects against Pseudomonas aeruginosa (FIC≤0.75), Acinetobacter baumannii (FIC = 1), and Escherichia coli (FIC≤0.75). The viability percentage of the cells after 24 h of exposure to a mixture of ZnO-NPs and SLA (about 50%) was more than when the cells were exposed to ZnO-NPs alone (about 30%) at the same concentration.

**Conclusions:**

A mixture of ZnO-NPs and CFS of probiotics can be an alternative to antibiotics, with more effectiveness and fewer side effects.

## 1. Background

Resistance to conventional antibiotics, especially in ESKAPE pathogens, has become a global health problem associated with high morbidity and mortality (1, 2). The acronym ESKAPE refers to a group of life-threatening nosocomial pathogens, including *Enterococcus faecium (E. faecium), Staphylococcus aureus (S. aureus), Klebsiella pneumonia (K. pneumonia), Acinetobacter baumannii (A. baumannii), Pseudomonas aeruginosa (P. aeruginosa), and Enterobacter spp. Enterobacteriaceae* has been added to ESKAPE bacteria according to the World Health Organization's (WHO) global priorities for drug-resistant pathogens (3). 

Nowadays, bacteriotherapy is used as one of the new alternatives against pathogenic organisms. In this method, probiotics, as beneficial and safe bacteria, are often used with or without their supernatant to treat or prevent infections (4, 5). Since there is no clear standard for applying probiotic formulations for antimicrobial therapy, it is needed to address existing gaps in this field. Considering the end of the golden era of antibiotic treatment, failure to cure severe infectious diseases is a sign of decreased efficacy of antibiotic therapy (6). The main solution to the current crisis is to utilize new alternatives and antibiotics that are both effective and safe against persistent infections (7, 8).

Cell-free supernatant (CFS) derived from probiotics is known as an important source of antimicrobial agents. Today, mainly *Lactobacillus* spp. and their supernatants are used in probiotics research. *Lactobacillus* spp. products contain inhibitory substances against bacteria, such as hydrogen peroxide, ethanol, bactericidal proteins, lactic acid, benzoic acid, bacteriocins, and short-chain fatty acids (SCFAs), including acetic, butyric, and propionic acids (9). *Lactobacillus* strains, especially *Lactobacillus plantarum* and *Lactobacillus acidophilus*, have an inhibitory effect on the growth of resistant pathogenic strains such as *S. aureus*, *E. coli*, *P. aeruginosa*, and *A. baumannii*(10-12).

Nanomaterials and metal oxide nanoparticles as antibacterial agents are promising in medicine. Among them, zinc oxide colloidal nanoparticles (ZnO-CNPs) have received much attention. In addition, ZnO-NPs have been introduced as GRAS (generally recognized as safe) by the U.S. Food and Drug Administration (13). The bactericidal and bacteriostatic properties of zinc nanoparticles are mainly related to the generation of Reactive Oxygen Species (ROS) production, including H_2_O_2_, hydroxyl radicals (OH-), and O_2_^-2^ (peroxide). ROS leads to cell inhibition or death through several mechanisms (14, 15).

Previous studies have shown that the electrochemical proton decay caused by organic acids in the CFS of *Lactobacillus* spp., as well as peroxidation of membrane lipids by H2O2, can alter the permeability of the cell membrane. Additionally, these processes prepare the cell to be receptive to other antibacterial agents, such as metal nanoparticles (16). Also, the antimicrobial effects of nanoparticles and CFS of *Lactobacillus* spp and the cytotoxicity effects of zinc nanoparticles have been investigated. However, the efficacy of a mixture of CFS of *Lactobacillus* spp and ZnO-NPs against ESKAPE strains has not been evaluated. Besides, the cytotoxic effects of the mixture of ZnO-CNPs and CFS of *Lactobacillus* spp have not been investigated. Therefore, we investigated the inhibitory and cytotoxic effects of ZnO-CNPs combined with the CFS of *L. plantarum* (SLP) or *L. acidophilus *(SLA) alone and in a mixture.

## 2. Objectives

This study investigated the antibacterial effect of nanoparticles and CFS of *Lactobacillus* spp and the cytotoxicity effects of zinc nanoparticles. However, the main aim was to investigate the synergistic effects of ZnO-CNPs combined with the CFS of *L. plantarum* or *L. acidophilus *concerning antibacterial activity and toxicity.

## 3. Methods

### 3.1. Bacterial Strains and Culture Conditions

Non-probiotic bacterial strains were ESKAPE, including *S. aureus* ATCC 25923, *E. coli* ATCC 25922, *P. aeruginosa* ATCC 27853, and *A. baumannii*ATCC 19606, obtained from the Iranian Biological Resource Center. Probiotic bacterial strains, including *L. plantarum* 299 V (DSM 9843) and *L. acidophilus *(LAFTI-L10 DSL), were obtained from the Iranian Biological Resource Center and the Dutch company DSM representative office in Iran (Iran Industrial Enzymes Company). The non-probiotic bacteria were cultured in Brain Heart Infusion (BHI) agar (MERCK, Germany) at 37°C for 18 h. *Lactobacillus* strains were cultured in deMan, Rogosa, and Sharpe (MRS) agar medium (Merck, Germany) under microaerophilic conditions at 37°C for 48 h (4). The fresh strains were preserved in the preservative medium and stored at - 20°C until use. Microbial confirmation and identification were performed by gram staining, microscopic and macroscopic observation, and standard biochemical tests, including Catalase, Oxidase, Simon citrate, Urea, and Triple Sugar Iron (TSI).

### 3.2. Preparation of Probiotics Cell-free Supernatant

To prepare probiotics CFS, *Lactobacillus* strains were cultivated in tubes containing MRS broth medium (MERCK, Germany) under microaerophilic conditions at 37°C for 24 h. The tubes were centrifuged (Sigma 3-16 k, Germany) at 10,000 g at 4°C for 30 min. Then, the supernatant was filtered through 0.22 μm filters (Millipore, Bedford, MA) (17).

### 3.3. Preparation of Zinc Oxide Nanoparticles

Zinc acetate dihydrate (ZnAc_2_, Zn (CH_3_COO)_2_.2H_2_O, Merck, purity 99%) was purchased as a commercial zinc source without further purification. Deionized water (DI) was used as a preparation solution. A typical synthesis procedure of ZnO nanopowders was performed as follows: The first reaction solution was obtained by dissolving 0.1 mol ZnAc2 in 15 mL of DI. Next, 27 mmol of Ammonium bicarbonate (NH_4_HCO_3_, Merck, purity 98%) was diluted in 28 mL of DI to make the second reaction solution. Subsequently, the first solution was dropwise added to the second solution. The final solution was stirred using a magnetic stirrer properly for 3 h. The mixed solution was centrifuged for 15 min at 5000 rpm. The white precipitates were washed thrice with DI and ethanol before being dried in a 70°C oven. Finally, desiccated products were annealed at 500°C for 3 h with a ramp rate of 7°C/min in a muffle furnace to produce ZnO nanopowder. The reactions related to the synthesis of ZnO-NPs are given below (18) (Equations 1 and 2)

Equation 1.


$${5zNaC}_{2}\;+\;{10NH}_{4}{HCO}_{3}\to Zn5{\left(OH\right)}_{6}{\left({CO}_{3}\right)}_{2}\left(s\right)+{10NH}_{4}Ac+{8CO}_{2}↑+{2H}_{2}O$$

Equation 2.


$$Zn5{\left(OH\right)}_{6}{\left({CO}_{3}\right)}_{2}\left(s\right)\overset{500{}^{\circ}C,\;3h}{\to }\;5ZnO\left(s\right)+2{CO}_{2}↑+3H_{2}O↑$$

To prepare a suspension of ZnO NPs, a probe ultrasonic homogenizer (Topsonics,

UHP-400 Model, Iran) was used to disperse 5 mg of ZnO powder into 50 mL of DI for 25 min at 200 W.

### 3.4. Characterization Techniques

**Figure 1.**
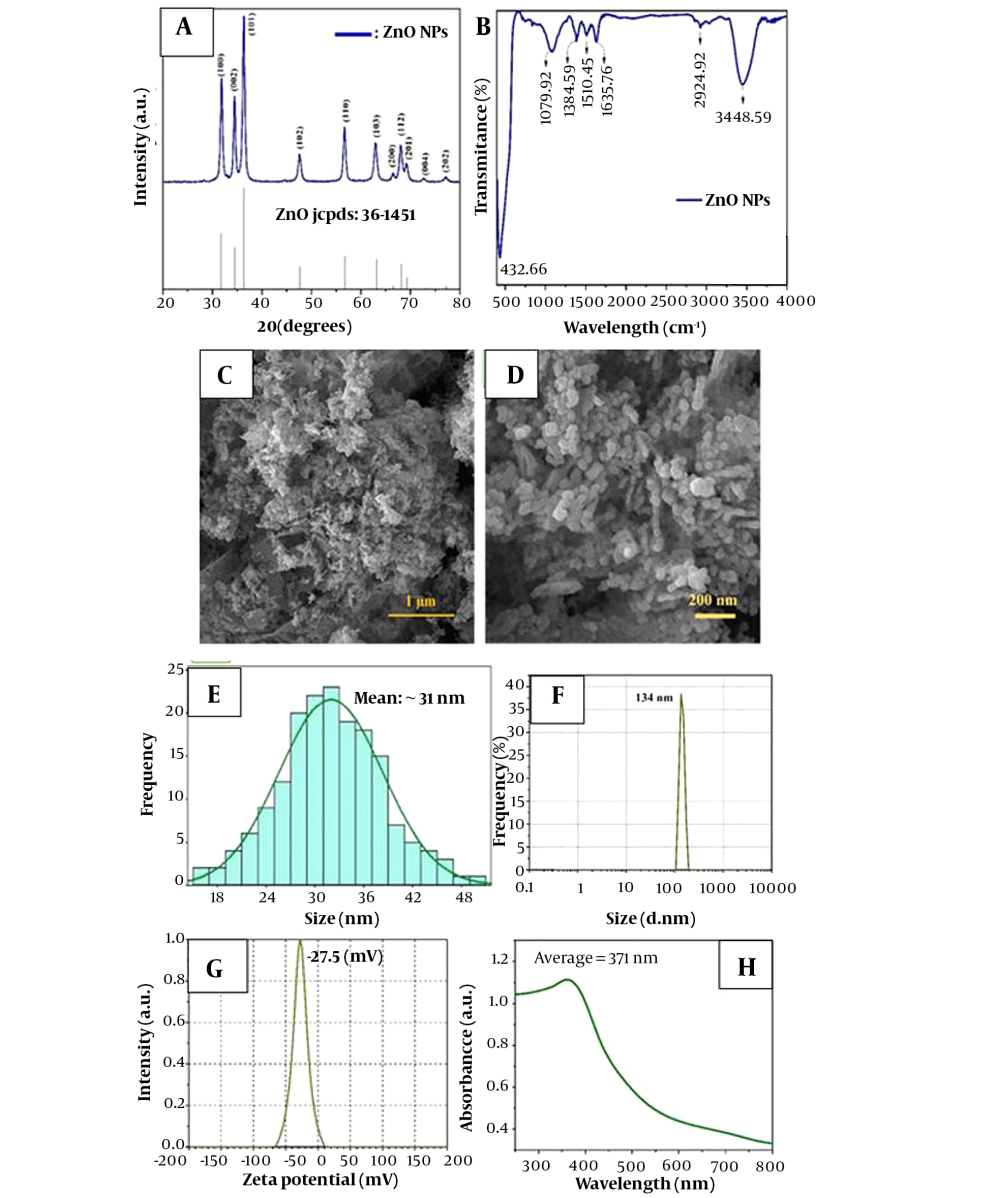
Characterization of ZnO-NPs nanoparticle: (A) X-ray diffraction pattern (XRD), (B) FTIR pattern, (C, D) FESEM micrographs, (E) particle size distribution of the synthesized sample, (F) DLS, (G) Zeta-potential, (H) UV-Vis spectra

The crystalline structure of ZnO-NPs was characterized by X-ray diffraction (XRD, PHILIPS PW1730) employing Cu-Kα radiation (λkα = 1.54 Å). Surface functional groups of synthesis samples were determined by Fourier Transform Infrared Spectroscopy (FTIR, 360 Nicolet AVATAR spectrometer, Thermo Scientific, USA). The FTIR analysis was performed to determine the chemical composition and functional groups of synthesized ZnO nanopowder under the infrared wavenumber of 400 - 4000 cm−1 (Figure 1B). 

Field-emission Scanning Electron Microscope (FESEM, MIRA3TESCAN-XMU) was used to observe the morphology and measure the size of nanoparticles. To determine the specific surface area and the porosity of synthesized ZnO-NPs, the Brunauer-Emmett-Teller (19) (BET, BELsorp-MINI II, BEL, Japan) technique was used to measure the adsorption isotherm of N2 gas molecules at a temperature of 77 K. The optical properties of the synthesized sample were studied using a UV–Vis spectrophotometer (UV-vis, AVaSpec 2048 TEC). The hydrodynamic size (100 μg·mL-1) and zeta potential of ZnO NPs were evaluated by Dynamic Light Scattering (DLS, SZ100 Model, Horiba, Japan) at room temperature.

3.5. Antibacterial activity of *Lactobacillus plantarum* and *Lactobacillus acidophilus *CFS and ZnO-CNPs

#### 3.5.1. Agar Well Diffusion Assay

The inhibitory effects of SLP, SLA, and ZnO-CNPs against microorganisms were determined by the agar well diffusion assay. The bacterial strains were grown in Mueller-Hinton (MH) broth (Merck, Germany) until they reached the 0.5 McFarland turbidity standards. Subsequently, 6 mm diameter wells were punched into MH broth (Merck, Germany) and filled with approximately 100 µL of ZnO-CNPs (at concentrations of 140 µg/mL), SLA, SLP (at concentrations of 100 µL/mL), and Double Distilled Water (DDW) as negative control. The agar plates were incubated at 37°C for 24 h. Then, the inhibition zone diameters were measured with a ruler. Three replications were used for each of the tests.

#### 3.5.2. Minimum Inhibitory Concentration Assay

The broth microdilution method in 96 well plates was used to determine the antibacterial agents' minimum inhibitory concentration (MIC). The working concentrations ranging from 50 µL/mL to 0.39 µL/mL were prepared from the stock solution (100 µL/mL) of CFS using DDW according to the Institute of Laboratory and Clinical Standards (CLSI) 2022 instructions. Dilutions of ZnO-CNPs, ranging from 70 - 0.54 µg/mL, were prepared from the stock solution (140 µg/mL) by incorporating ZnO-CNPs into DDW. The total volume considered for each well was 200 µL/mL. For each test, 100 µL/mL of MH broth medium (Merck, Germany) was added to each well of a 96-well Microtiter Plate. Then, 100 µL/mL of each agent was added, and serial dilutions were prepared. The bacterial suspension containing approximately 106 Colony-forming Units (CFU)/mL was prepared from a 24-hour culture and added to all wells except negative control. Finally, the microtiter plates were incubated at 37°C for 24 h. The concentration completely inhibiting bacterial growth (first clear well) was considered the MIC.

#### 3.5.3. Minimum Bactericidal Concentration Assay

The minimum bactericidal concentration (MBC) shows the lowest concentration, killing 99.9% of the inoculated bacteria after 18 - 24 h incubation at 37°C. For this purpose, 100 µL/mL of the well content of the MIC and two higher wells were taken and spread on MH agar medium. The concentration of samples with less than 10 colonies was considered the MBC.

#### 3.5.4. Fractional Inhibitory Concentration (FIC) and Interaction Effect of Two Antibacterial Agents

Drug interactions (ZnO-NPs with SLP or ZnO-NPs with SLA) were evaluated using the Checkerboard test. The modified and simplified Checkerboard test was performed using the method described by Bellio et al. In the Checkerboard method, broth micro-dilution assay was performed in a 96-well plate with a final volume of 200 μl for each strain. Mueller–Hinton Broth medium (90 µL) was dispensed to wells. Drug A (ZnO-NPs) was prepared 4-fold more concentrated than the MIC (alone), and 90 μL of it was inoculated into rows A-E from column 1. Then, drug A was diluted from left to right up to 4 dilutions lower than the MIC (alone). After that, 2-fold higher and 2-fold lower than the MIC of drug B (SLA or SLP) were prepared, and 90 µL of each dilution was inoculated into the wells without dilution to produce the same drug B dilutions in each row. Bacterial inoculation (106 CFU/mL) was performed into each well. The microplates were placed in the incubator at 37ºC for 18 to 20 h (20). This test used the mixture of two compounds to obtain combined concentrations of two compounds. For the drug interactions assessment, Fractional Inhibitory Concentration (FIC) Index (FICI) was calculated using the following formula:


$$FIC\;index\;=\;F\frac{ICA\;+\;FICB\;=\;MIC\;(A\;in\;the\;presence\;of\;B)}{MIC\;(A\;alone)}+\frac{\;MIC\;(B\;in\;the\;presence\;of\;A)}{MIC\;(B\;alone)}$$

According to this method, the four categories based on the FICI are defined as follows:

synergism: FIC ≤ 0.5; additivity: 0.50 < FIC ≤ 1; indifference: 1 < FIC ≤ 4; antagonism: FIC > 4 (20)

### 3.6. Cell Culture

The Hu02 cell line (human normal fibroblast cell, IBRC C10309) was cultured in a T25 flask containing Dulbecco's Modified Eagle Medium (DMEM), high glucose (Gibco, USA) with Fetal Bovine Serum (FBS) 10% (Gibco, USA), 100 µg/ mL penicillin and 100 µg/ mL streptomycin (Sigma, USA) in an incubator (37°C, 5% CO_2_, and 95% humidity) for 24 - 48 h.

### 3.7. Cytotoxicity Assay

The MTT assay measured cellular metabolic activity to indicate cell viability, proliferation, and cytotoxicity for cytotoxicity testing. We obtained 3-(4,5-Dimethyl-2-thiazolyl)-2,5-diphenyl-2H-tetrazolium bromide (MTT) from Sigma-Aldrich (USA). The cells were passaged and trypsinized. The cell suspension (104 cells/ mL) was prepared and added into micro-wells of 96 wells containing the mentioned culture medium and incubated for 24 h. Later, freshly prepared SLA and SLP at various concentrations (1/4 MIC,1/2 MIC, MIC, 2 MIC: From 3.125 to 25 µL/mL) and freshly prepared ZnO-CNPs at various concentrations (1/16 MIC, 1/8 MIC, 1/4 MIC, 1/2 MIC, MIC, 2 MIC: From 2.18 to 140 µg/mL) were added to the wells and incubated for 24, 48, and 76 h (37°C, 5% CO_2_). Next, an MTT assay was performed for the mixture of both agents with a synergistic effect according to the FIC test (with a certain concentration in a 1: 1 ratio). After that, 20 μL of MTT solution (5 mg of MTT in 1 mL PBS) was added to each well, and the microtiter plate was incubated for 4 h more at dark (37°C). The supernatant was discarded, and 150 μL of DMSO (Dimethyl Sulfoxide) was added to each well. The microplates were shaken with the shaker (Labnet Orbit P4, USA) for 5 to 10 minutes. After formazan crystals dissolved, the absorbance of the well content was determined spectrophotometrically at the wavelength of 570 nm using a microplate reader (Biotech, elx800, USA). This experiment was repeated three times. The viability percentage was measured according to the below formula (21).

%viability: (absorbance sample / average absorbance negative control) ×100

### 3.8. Statistical Analysis

Excel 2019 and SPSS. 28 software were used to analyze the data. Descriptive statistics, including central tendency indices (mean and standard deviation), were used as graphs. The mean differences were investigated through one-way ANOVA and Tukey's post hoc test.

## 4. Results

### 4.1. Synthesis and Chemistry

Figure 1A illustrates the XRD pattern of synthesized ZnO-NPs, which is identical to the single-phase ZnO with a hexagonal structure. Based on the Debye-Scherrer formula (22), particle size was in the range of 28 - 43 nm.

Comparing the synthesized ZnO pattern with the standard pattern, the intensity and position of diffracted peaks that are well-matched with the standard pattern confirm the high purity of the synthesized nanopowders (22).

According to the FTIR analysis (Figure 1B), the characteristic peaks of ZnO were observed at

the wavelengths of 432, 1384, and 1635 cm-1, all of which match the stretching vibration of ZnO (23).

The surface morphology and size details of synthesized ZnO-NPs are shown in Figure 1C-E. According to the FESEM images, an average particle size of 31 nm for ZnO-NPs is observed. Also, ZnO-NPs have a homogeneous distribution of spherical particles, which agrees with Scherrer's formula results utilizing XRD data (23).

DLS analysis (Figure 1F) shows that the average hydrodynamic size of ZnO-NPs was about 134 nm. Because of the Brownian motion of NPs in suspension, the particle size acquired by DLS was substantially larger than that obtained by FESEM (23, 24).

As shown in Figure 1G, it can be seen that the average zeta potential of ZnO-NPs in water was - 27.5 mV.

UV–Vis spectroscopy was used in the range of 250 to 700 nm to further investigate the ZnO-NP electronic structure. As shown in Figure 1H, the characteristic strong band and the absorption edge of ZnO were displayed at the wavelength of about 371 nm and 521 nm, respectively.

### 4.2. Antibacterial Activities

**Figure 2.**
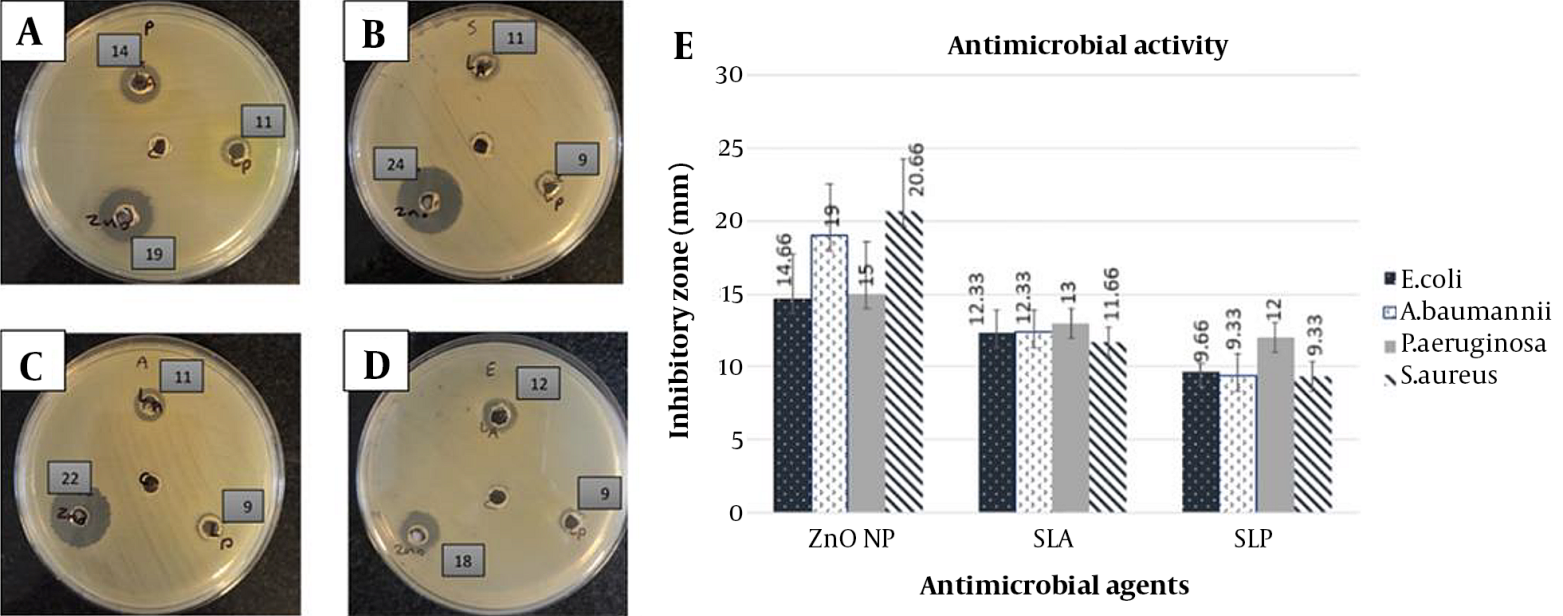
Antibacterial activity (agar well diffusion assay): (A-D) Inhibition zone of antibacterial agents on Muller-Hinton agar medium. ZnO-CNP at a concentration of 140 µg/mL (down and left), SLP at a concentration of 100 µL (down and right), SLA at a concentration of 100 µL (up) against *P. aeruginosa* ATCC 27853 (A), *S. aureus* ATCC 25923 (B), *A. baumannii*ATCC 19606 (C), and *E. coli* ATCC 25922 (D). The well in the middle of the plate: DDW as negative control. The numbers beside the wells: Inhibition zone of antibacterial agents. (E) Mean inhibitory diameter of antibacterial agents against ESKAPE bacterial strains (mean ± SD). Abbreviations: SLA, CFS of *Lactobacillus acidophilus*; SLP: CFS of *Lactobacillus plantarum*; ZnO NP: ZnO-CNPs

The antibacterial activity of SLA, SLP, and ZnO-CNPs against ESKAPE strains, including *P. aeruginosa* ATCC 27853, *S. aureus* ATCC 25923, *A. baumannii*ATCC 19606, and *E. coli* ATCC 25922 was determined by the agar well diffusion assay. Here, DDW was used as a negative control (Figure 2). Figure 2A-D shows the inhibition zone of agents, and Figure 2E shows the mean inhibitory diameters of agents (mean ± SD). Data analysis was performed by the ANOVA statistical test, followed by the Tukey post-hoc test. Based on the inhibitory zones, the effect of ZnO-CNP against *S. aureus* was significantly stronger than that of SLA (P = 0.005) and SLP (P = 0.002). Also, its inhibitory effect against *A. baumannii*was significantly stronger than that of SLA (P = 0.035) and SLP (P = 0.007). In addition, the inhibitory effect of ZnO-CNP against *E. coli* was significantly stronger than that of SLP (P = 0.05).

The MIC and MBC of antibacterial agents against all used ESKAPE strains are shown in Table 1. Accordingly, the MIC of SLP was lower than that of SLA against *P. aeruginosa* and *S. aureus*. In addition, the MIC of ZnO-NPs was lower against *S. aureus* and *E. coli* than against *P. aeruginosa* and A. baumannii.

**Table 1.** The Minimum Inhibitory Concentration (MIC) and the Minimum Bactericidal Concentration (MBC) for Three Antibacterial Agents Against Four Tested ESKAPE Strains

Agent	SLA µL /mL		SLP µL /mL		ZnO-NPs µg/ mL	
	MIC	MBC	MIC	MBC	MIC	MBC
Microorganism						
*S. aureus* ATCC 25923	12.5	25	6.25	12.5	35	70
*P. aeruginosa* ATCC 27853	12.5	12.5	6.25	6.25	70	140
*A. baumannii* ATCC 19606	6.25	12.5	6.25	6.25	70	140
*E. coli* ATCC 25922	12.5	12.5	12.5	25	35	35

Abbreviations: SLA, CFS of *Lactobacillus acidophilus*; SLP, CFS of *Lactobacillus plantarum*; ZnO-NPs, ZnO-CNP.

The MIC and MBC results were used in the Checkerboard test (Table 2). As shown, the interaction effects of antibacterial agents, including ZnO-NPs, combined with SLA or SLP, were investigated. All combined antibacterial agents in the current study demonstrated synergistic and additive effects against *P. aeruginosa*, A. baumannii, and *E. coli*, but they had an indifferent effect against *S. aureus*.

**Table 2.** The Fractional Inhibitory Concentration (FIC) and Interaction Effect of Two Antibacterial Agents (ZnO-NPs and SLP or ZnO-NPs and SLA) Against ESKAPE Strains

Microorganism and Combination of Two Compounds	MIC A, (Alone), µg/ mL	MIC A (in the Presence of B), µg/ mL	MIC B, (Alone), µL /mL	MIC B (in the Presence of A), µL /mL	Chekerboard FIC Index	Chekerboard Effect
***P. aeruginosa* ATCC 27853**						
ZnO-NPs and SLA	70	17.5	12.5	3.125	0.5	S
ZnO-NPs and SLP	70	17.5	6.25	3.125	0.75	A
***A. baumannii* ATCC 19606**						
ZnO-NPs and SLA	70	35	6.25	3.125	1	A
ZnO-NPs and SLP	70	35	6.25	3.125	1	A
***E. coli*** ** ATCC 25922**						
ZnO-NPs and SLA	35	8.75	12.5	3.125	0.5	S
ZnO-NPs and SLP	35	8.75	12.5	6.25	0.75	A
***S. aureus*** ** ATCC 25923**						
ZnO-NPs and SLA	35	35	12.5	6.25	1.5	I
ZnO-NPs and SLP	35	35	6.25	3.125	1.5	I

Abbreviations: SLA, CFS of *Lactobacillus acidophilus*; SLP, CFS of *Lactobacillus plantarum*; ZnO-NPs, ZnO-CNP; agent A, ZnO-CNP; agent B, SLP or SLA.

The use of ZnO-CNP combined with SLA or SLP improved the inhibitory effect against *P. aeruginosa*, *A. baumannii*, and *E. coli* strains and had additive (FIC = 0.75 and 1) or synergistic (FIC = 0.5) effects. Thus, the MIC of agents in the presence of another agent was reduced by one-half or one-fourth of the individual use. However, their simultaneous use against *S. aureus* did not increase antibacterial activity (FIC = 1.5).

### 4.3. Cytotoxicity Assay

**Figure 3.**
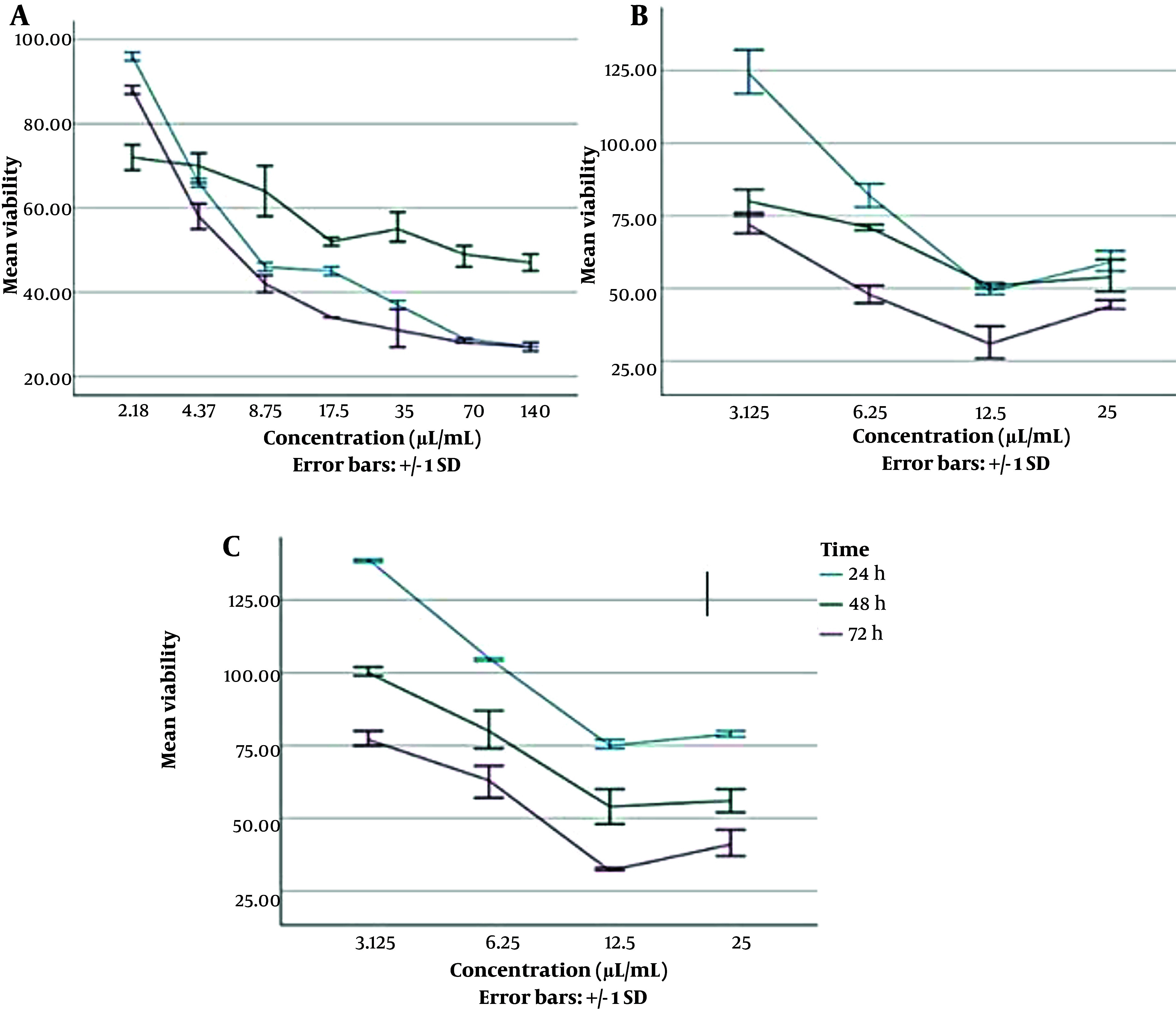
Viability percentage of Hu02 cells in the presence of different concentrations of antibacterial agents at different time intervals of 24,48 and 72 h (mean ± SD). (A) The diagram on the left presents ZnO-CNP at concentrations of 2.18 to 140 µg/mL; (B) The diagram in the middle presents SLA at concentrations of 3.125 to 25 µL/mL; (C) The diagram on the right presents SLP at concentrations of 3.125 to 25 µL/mL

We observed almost a decreasing trend of cell viability with increasing concentrations of agents at all three incubation times (Figure 3). However, the results demonstrated that the mean viability percentage of Hu02 cells exposed to SLP was significantly higher than when the cells were exposed to ZnO-CNP after 24 h (P = 0.012 by ANOVA and Tukey's post hoc test).

The mean viability percentage of cells exposed to agents in different concentrations after 24 h was as follows: SLP (99.89% ± 27.05) > SLA (79.24% ± 30.88) > ZnO-NPs (53.48% ± 23.2). According to Table 3, the cytotoxic effects were investigated for combined agents, which had synergistic effects based on the FIC test. The first mixture was ZnO-NPs with a concentration of 35 µg/mL and SLA with a concentration of 12.5 µL/mL, and the next mixture was ZnO- NPs with a concentration of 70 µg/mL and SLA with a concentration of 12 µL/mL (Table 3). 

**Table 3.** Viability Percentage of Hu02 Cells After 24,48 and 72 h of Incubation with Combined Agents (at a 1: 1 Ratio)

Antibacterial Agents	Viability% After Exposure Times (Hour)		
	24	48	72
**ZnO-NPs (35 µg/mL) and SLA (12.5 µL/mL)**	57.85 ± 0.36	70.37 ± 3.48	30.94 ± 0.46
**ZnO-NPs (70 µg/mL) and SLA (12.5 µL/mL) **	49.73 ± 0.37	67.11 ± 4.08	30.39 ± 0

Abbreviations: SLA, CFS of *Lactobacillus acidophilus*.

The viability percentage of the mixed agents was compared with the agents alone at the same concentrations. The ANOVA test and Tukey's post hoc test results demonstrated that the viability percentage of the cells was higher after 24 h of exposure to a mixture of ZnO-NPs (70 µg/mL) and SLA (12.5 µL/mL) compared to exposure to ZnO-NPs with the same concentration alone (P = 0.03). Also, the viability percentage of cells was higher after 24 h of exposure to a mixture of ZnO-NPs (35 µg/mL) and SLA (12.5 µL/mL) compared to exposure to ZnO-NPs with the same concentration alone (P = 0.01).

## 5. Discussion

The antibacterial effect of ZnO-NPs and CFS of *Lactobacillus* spp has already been proven, but their interaction effect has not yet been investigated. Therefore, in the present study, we investigated the interaction effects of ZnO-CNPs combined with the CFS of *L. plantarum* or *L. acidophilus*. In the current study, we observed the inhibition zones of all three agents used in this study against ESKAPE strains. The inhibitory effect of ZnO CNP against the ESKAPE strains was more than that of SLA and SLP. Metal and metal oxide nanoparticles show strong antibacterial activity even at extremely low concentrations. In fact, ZnO-NPs have antibacterial activity against a wide range of Gram- positive and Gram-negative bacteria. The production of ROS due to ZnO-NPs leads to the destruction of the cell wall, increased membrane permeability, and inhibition or death of bacterial cells. Properties such as high stability, the ability to change surface characteristics, and the high penetration power of ZnO-NPs (14) can justify their greater inhibitory effect compared to SLA and SLP in this study. We observed the inhibitory effects of SLA and SLP against ESKAPE strains. In this regard, Venosi et al. found that probiotic therapy has an inhibitory effect against Gram-positive and Gram-negative bacteria (25). Many studies have reported that the extracellular products of *Lactobacillus* spp have significant inhibitory activities against clinical isolates (17). The CFS of *Lactobacillus* spp contains antibacterial agents such as lipoteichoic acid, acetic acid, diacetyl, lactic acid, and hydrogen peroxide (26). Our study findings showed that the simultaneous use of ZnO-CNP and SLA or SLP had an additive or synergistic effect against the ESKAPE strains except for *S. aureus*. In fact, synergistic testing was used to determine whether two antibacterial agents are better to be used alone or in a mixture. An additive effect may help reduce the dose of each agent and may reduce side effects and adverse therapeutic effects (27). Consistent with our study, Ohirchuk and Kovalenko stated that *Lactobacillus* gasseri enriched with metal nanoparticles had a synergistic effect (28). In addition, Zheng et al. reported that ZnO nanoparticles combined with *L. plantarum* BLPL03 had a synergistic effect on the inhibition of pathogens (29). However, we did not find a study investigating the simultaneous use of probiotic supernatant and metal nanoparticles for their antibacterial properties. Organic acids secreted in the CFS of *Lactobacillus* spp collapse the electrochemical proton, and H_2_O_2_ secreted in the CFS peroxidizes the membrane lipids. This changes the permeability of the cell membrane and, as a result, disrupts the transport system of the membrane (16). Lactic acid acts as an antimicrobial compound by lowering the PH and also prepares the bacterial cell to accept antibacterial agents. In the same vein, it makes the membrane permeable in gram-negative bacteria, so the outer membrane of these bacteria may act as an enhancer for other antimicrobials (16). In the current study, the mean viability percentage of Hu02 cells exposed to SLP was significantly higher than that of cells exposed to ZnO-CNP after 24 h. In fact, the CFS of probiotics in the current study was not toxic at some concentrations for 24, 48, and 72 h. Almost the same finding was reported by Nehal M. El-Deeb et al., where they found that *L. acidophilus* 20079 metabolites had no toxic effect on normal epithelial cells (30). In this regard, Dolati et al. reported that the cytotoxic effect of Bacillus coagulans supernatant was only 23% on the Human Foreskin Fibroblast (HFF) cell line (31).

In the current study, we also observed the reduction of cells at some concentrations. Probiotic bacteria supernatant contains organic acids and exopolysaccharides. Metabolites of probiotics can induce apoptosis by upregulating pro-apoptotic genes and downregulating anti-apoptotic genes. Therefore, metabolites produced by probiotics may affect cell proliferation by induction apoptosis (32, 33). This content can explain the fluctuation in the curve of cell viability percentage over time in this study.

On the other hand, the toxicity of nanoparticles highly depends on cell type, concentration, and exposure time, irrespective of synthesis method. Increased ROS levels cause severe damage to the DNA of the cells, leading to cell cycle arrest and cell death (34). Also, the synergistic effect may help reduce the dose of each agent and consequently reduce toxicity, side effects, and adverse therapeutic events (27), consistent with the findings of our study. The viability percentage of cells exposed to a mixture of ZnO-NPs and SLA was higher than that of cells exposed to ZnO-NPs alone.

### 5.1. Conclusions

The present study indicated that a mixture of ZnO-NPs and SLA or SLP improved the inhibitory effect against ESKAPE strains compared to using each agent alone. In addition, the Hu02 cell viability percentage was higher after 24 h of incubation with a mixture of ZnO-NPs and SLA than when nZnO was used alone. Therefore, using a mixture of ZnO-NPs and SLA or SLP may be a good candidate to overcome nosocomial infections with more effectiveness and fewer side effects. Of course, more extensive studies are needed to make a more definitive decision.

## Data Availability

The data underlying this article will be shared on reasonable request to the corresponding author.
